# Percutaneous Repair of Chronic Aortic Pseudoaneurysm: A Single-Center Experience

**DOI:** 10.1016/j.jscai.2024.102249

**Published:** 2024-08-09

**Authors:** Bruce E. Lewis, Dominick V. Bufalino, Mohammed H. Hussein, Sorcha Allen, Lukas Burke, Rashad J. Belin, Marc G. Henderson, Jeffrey Schwartz

**Affiliations:** a Division of Cardiovascular Medicine, Loyola University Medical Center, Maywood, Illinois; b Department of Internal Medicine, Ascension Saint Joseph Hospital, Chicago, Illinois; c Cardiac Catheterization Laboratories, Loyola University Medical Center, Maywood, Illinois; d Department of Thoracic and Cardiovascular Surgery, Loyola University Medical Center, Maywood, Illinois

**Keywords:** Amplatzer, occluder, percutaneous, plug, pseudoaneurysm

## Abstract

**Background:**

Aortic pseudoaneurysm (AP) is a late complication of aortic repair that, without intervention, carries a high mortality rate. Surgical repair has significant in-hospital mortality and high health care costs. Endovascular stent grafting use is currently limited to branch-free aortic segments or the use of complex fenestrated devices. Our objective was to review the literature and share our institution’s experience with AP percutaneous closure by vascular plugs and occluder technology.

**Methods:**

We retrospectively reviewed percutaneous AP closure cases published in the literature (2005-2016) and from our institution (2017-2019). The follow-up strategy in our institution group was up to the discretion of the performing physician. We measured the procedure’s safety, complications, and follow-up outcomes.

**Results:**

We found 40 cases in the literature and 10 at our institution. The procedural success rate was 90% in the literature and 100% in our group. Our group’s average length of stay was 1.9 days with no observed major procedural complications. The literature’s follow-up was generally limited to the hospitalization period. Our patients had a median follow-up time of 12 months (range 3-47 months). Late follow-up of AP demonstrated that sac size remained stable or reduced in 6 patients, but a size increase was observed in 4 patients requiring surgical intervention. Death from nonprocedure-related complications occurred in 40% of our patients. The cost per procedure was hypothetically less than for performing open surgical repair.

**Conclusions:**

Our experience shows a viable option for percutaneous AP repair, given its initial safety and cost-effectiveness. Our experience highlights the critical role of follow-up imaging in identifying AP expansion and the need for further intervention. The high nonaorta-related mortality seen in follow-up emphasizes the high-risk nature of the population due to comorbidities.

## Introduction

Sir William Osler noted, “There is no disease more conducive to clinical humility than aneurysms of the aorta.”^1^ Cardiac and vascular surgery approaches to aortic pathology have evolved over the past 5 decades, yet despite continued improvement in surgical and endovascular techniques, long-term follow-up occasionally detects late complications from initially successful repairs. One such feared complication is pseudoaneurysm formation, most seen at sites of anastomosis, cannulation, or cross-clamping of the aorta.

Medical treatment of aortic pseudoaneurysm (AP) carries a published mortality of up to 61%.^2^ The traditional approach of surgical repair has been quite effective in reducing long-term mortality but carries an in-hospital mortality of 6.9% to 12.6%,^3^ extended recovery, high cost to the health care delivery system, and substantial challenges for the surgical team addressing these patients' problems. The “redo” nature of these cases also adds to the surgical complexity. The significant mortality observed with traditional surgical approaches and the known poor outcomes previously reported with conservative management justify an investigation into alternative endovascular technologies. Endovascular stent grafting would appear to offer an attractive alternative to open repair. However, this technology is associated with an all-cause mortality of 15.2%, aorta-related mortality of 5%, a type I endoleak rate of 18.6%, and a 9.3% reintervention rate.^4^ Furthermore, traditional endograft repair is limited to “branch-free” aortic segments or the use of complex fenestrated devices. These observations support the need for an effective, safer, and less complicated approach to pseudoaneurysm management. Interest in new applications of vascular plug and vascular occluder technology may offer a safe, effective, and durable solution for these challenging cases.

Our surgical and interventional group has developed a particular interest in aortic pathology, which has resulted in referrals for patients with postsurgical AP formation. The purpose of this manuscript is to review the published literature pertaining to a nonsurgical approach to pseudoaneurysm repair of chronic, noninfectious pseudoaneurysms. We would also like to outline our experience and share technical considerations that can optimize success, enhance safety, and minimize costs to the health care delivery system. Finally, we would like to create reasonable expectations of the outcomes of this technology in terms of procedural success, morbidity, mortality, and durability.

## Methods

### Literature review

For this review, we searched PubMed, Embase, and Google Scholar databases in English with no data restriction using search terms related to AP and repair cases. We reviewed the literature for the following terms: septal occluder device, Amplatzer, occluder, atrial septal defect (ASD), false aneurysm, and pseudoaneurysm. Our literature search yielded 151 citations that were then reviewed. We identified a total of 378 cases of AP in the literature. Seventy-four percent of these cases were repaired surgically, 21% were treated with endovascular techniques (including stent graft placement and septal occluder device implantation), and 5% were considered too high risk for intervention.^5^ We isolated 28 publications covering 40 cases of percutaneous AP closure using varied techniques, including plug and occluder devices, in addition to coil embolization. These publications were reviewed for patient characteristics, anatomic dimensions of the pseudoaneurysm, procedural approaches, and clinical and imaging follow-up.^5^, ^6^, ^7^, ^8^, ^9^, ^10^, ^11^, ^12^, ^13^, ^14^, ^15^, ^16^, ^17^, ^18^, ^19^, ^20^, ^21^, ^22^, ^23^, ^24^, ^25^, ^26^, ^27^, ^28^, ^29^, ^30^, ^31^

### Data collection

We retrospectively reviewed the catheterization laboratory database at our tertiary care institution (Loyola University Medical Center) for cases of percutaneous AP repair that were referred by cardiovascular surgery for a percutaneous option and patients deemed by cardiovascular surgery to be at very high risk for open surgical repair. We identified 10 cases of nonstent endovascular treatment that took place between March 2017 and April 2019. The electronic medical record and radiology archiving system were accessed, and anonymized data were collected. Patient demographic characteristics, imaging studies, and procedural data were all recorded in addition to data from follow-up visits and clinical outcomes. Information about the patient's sex was collected from the electronic medical records and represents the assigned sex at birth. Postprocedural bleeding was defined using the Bleeding Academy Research Consortium scale. The standard of care for these patients at our institution includes frequent office visits and imaging follow-ups as part of their postprocedural care. Mortality events and causes of death were obtained through medical records review and follow-up telephone calls. Ethical approval was obtained from the institutional review board of Loyola University Medical Center prior to project commencement.

### Statistical analysis

The mean was used to measure the average length of hospital stay, while the median was measured for the follow-up duration. Percentages were taken for the rates of successful device delivery, worsening postprocedural AP sac size, postprocedural major bleeding, surgical interventions, in-hospital, and all-cause mortality. Correlation analysis between the 2 groups was not conducted due to the low sample size, the unmatched baseline characteristics, and the limited number of cases. Analysis was performed using JMP 16.0 (SAS Institute).

### Technical observations

**Figure 1:**
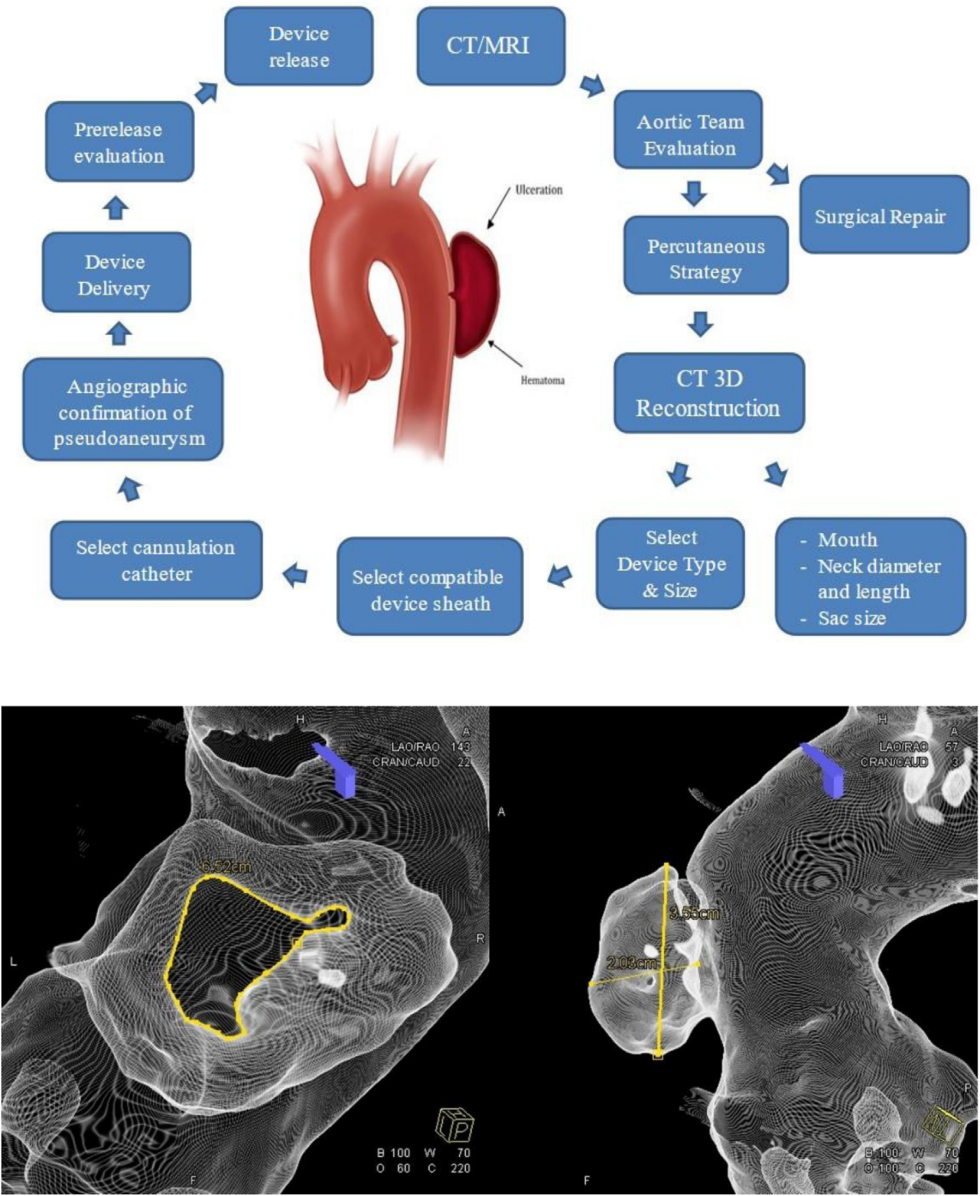
**Our institution's procedural approach and some imaging techniques used for the percutaneous aortic pseudoaneurysm (AP) repair.** The flowsheet on top outlines our procedural approach steps to percutaneous AP repair. The 2 images at the bottom show an example of our utility of 3-dimensional computed tomography (3D CT) reconstruction in measuring the AP mouth perimeter (bottom left) and AP sac dimensions (bottom right). MRI, magnetic resonance imaging.

Successful vascular plug/occluder deployment and closure of an AP requires thoughtful preprocedural planning and a multidisciplinary approach, including the participation of cardiothoracic surgeons, interventional cardiologists, and cardiac imagers to evaluate a patient's candidacy for the procedure. A thorough knowledge of the variety of vascular plugs and occluder devices available is essential to maximize procedural success. Review of the product catalogs to guide equipment selection can eliminate unnecessary procedural steps by ensuring device and delivery sheath/catheter compatibility. Figure 1 outlines our approach to each case, highlighting the team approach and the many steps involved.

### Preprocedural planning

All patients underwent preprocedural contrast-enhanced computed tomography (CT) of the thoracic aorta with post hoc 3-dimensional (3D) reconstruction. All 3D reconstructions were performed by 2 dedicated radiology technicians. The following measurements were obtained: pseudoaneurysm sac size; long axis, short axis, and perimeter of the pseudoaneurysm mouth; diameter and length of the pseudoaneurysm neck. Figure 1 demonstrates our use of 3D reconstruction techniques in CT imaging to measure AP dimensions. These measurements were then reviewed by the procedural team to determine device selection.

Preprocedure measurements of device size were then compared to the initial outcome and follow-up imaging. The partial success seen in a later case prompted a retrospective evaluation of pseudoaneurysm mouth perimeter measurements. Perimeter or occluder waist dimensions were obtained from the manufacturer. The perimeter of the occluder plug waist selected could then be compared to measurements of the pseudoaneurysm mouth perimeter to assess a potential enhancement in procedural success. A comparison of preprocedure pseudoaneurysm neck dimension to plug occluder size and a CT/MR follow-up assessment was done to identify the optimal strategy needed to guarantee long-term success.

### Pseudoaneurysm dimension measurement and device selection

The adage “one size fits all” does not apply to percutaneous pseudoaneurysm repair. Device selection is initially determined using preprocedural imaging and can be modified using intraprocedural cine angiography. Plug sizes are dictated by the largest diameter ideally measured using 3D reconstruction of the pseudoaneurysm mouth. We recommend a plug upsizing of approximately 20% in more circular-shaped pseudoaneurysm mouths. Sizing of the device waist to the true long axis in eccentric mouths may lead to oversizing and incomplete disc apposition on the aortic wall. Device selection in patients with marked discrepancies between the long axis and short axis length of the pseudoaneurysm mouth may be better approached with sizing to the true perimeter of the pseudoaneurysm mouth. The length of the neck helps to determine plug type. Longer necks allow for safe delivery of vascular plugs whereas shorter necks with wide mouths may be better treated with ASD and ventricular septal defect (VSD) occluder devices, utilizing the dual disc construction to secure the plug reliably. An important principle to understand when using occluder devices is the reliance on the occluder device's central waist size to close the defect and the use of the outer discs to anchor the device. Very small diameter necks may require balloon predilation to allow for safe entry of the delivery sheath or catheter into the pseudoaneurysm. Careful selection of balloon size can ease sheath delivery and reduce the probability of dissection and trauma to the aorta during delivery.

Larger mouth pseudoaneurysms with shorter neck lengths are generally approached with ASD, patent foramen ovale, and VSD-type occluders which allows fixation of the plug and prevents subsequent embolization. Patent foramen ovale devices have no significant waist diameter, which will not effectively occlude the pseudoaneurysm neck and depend on disc apposition with subsequent endothelialization to exclude the aneurysm. We rely heavily on the Amplatzer catalog to appropriately select device size and geometry. Low-pressure balloon occlusion of the pseudoaneurysm neck can provide confirmation of neck mouth diameter. The technique used for ASD sizing can also be applied to neck pseudoaneurysm assessment using contrast-enhanced fluoroscopic measurement of the balloon waist or ultrasound demonstration of flow elimination and measurements of balloon diameter. Color flow assessment with intracardiac echocardiography offers a more sensitive flow assessment than cine angiography.

### Delivery sheath selection

The selection of the delivery conduit (sheath or guide catheter) is dictated by the preprocedure plug/occluder device selection. Large pseudoaneurysms that require ASD or VSD occluders necessitate 9F or 10F delivery sheaths and represent the biggest challenge in delivery management. The standard Amplatzer sheath for ASD closure has a maximum length of 80 cm which may be insufficient to reach aneurysm sacs in the proximal ascending aorta of tall patients (5 feet 10 inches and taller). Ten F sheaths longer than 80 cm are not currently available in the United States. Measurement of the external catheter from the skin to the catheter hub can provide a reasonable estimate of the probability of successful sheath cannulation of the pseudoaneurysm with the 80 cm sheath. In taller patients, a 12F 94-cm sheath can be used at the expense of upsizing to 12F arterial access. Some operators have overcome the length limitation of the standard 80 cm Amplatzer sheath with a brachial approach, but the brachial approach carries the obvious concern of vascular site complications with a 9F or 10F entry in the smaller caliber brachial artery.

### Pseudoaneurysm cannulation

Preprocedural reconstruction of each aorta using a 3D printer was extremely valuable in catheter selection. The patient’s reconstructed model of the aorta could be used to identify the family of catheters that would effectively cannulate the pseudoaneurysm. This technique markedly reduced fluoroscopy and procedural times and resulted in increased efficiency of the procedure. Standard 6F coronary diagnostic or guide catheters appear to provide an effective tool for the location of the pseudoaneurysm neck. Small pseudoaneurysms that require vascular plugs can be directly cannulated with a guide catheter. Larger pseudoaneurysms that require occluder devices are usually cannulated with diagnostic catheters inserted through the larger sheath, which is then used to deliver the occluder device. The 6F Judkins Right 4 diagnostic catheter shape was most used for neck cannulation in our institution. Pseudoaneurysm necks with acute angles were successfully cannulated with 6F internal mammary catheters. Necks with inferior takeoff were typically cannulated with multipurpose catheters or hockey stick guide catheters. Very small pseudoaneurysm necks can be approached with a 5F guide catheter delivery system “telescoped” through a 90-cm guide catheter. The Cordis catheter provides enough support to gain the necessary maximum wire purchase in the pseudoaneurysm sac.

### Wire selection

Pseudoaneurysms with large mouth sizes, short neck lengths, and acute angulations provide the biggest challenge to the successful delivery of the closure device. These procedures require maximum wire support to facilitate large sheath delivery to accommodate the occluder family of devices. We have utilized 3 approaches to provide adequate support for sheath delivery into the aneurysm sac when presented with challenging anatomy. The first approach is a standard 0.035-inch stiff Amplatz with a 7.5 cm “soft tip,” which usually provides adequate support for the delivery of large sheath sizes. When challenged by acute angles, a “coiled wire” approach with a more friendly wire (eg, Wholey and Benston) can allow a safer and less traumatic entry of the telescoped guide catheter into the sac. Once optimal wire position has been achieved, a 6F diagnostic catheter can be used inside the large sheath and advanced over the wire deep into the pseudoaneurysm sac to provide additional “rail” support and facilitate sheath advancement. A more flexible and “slippery coated” sheath would certainly be preferred if the option exists. In cases where the pseudoaneurysm has 2 mouths, we use a soft wire advanced from one mouth out through the second mouth. The wire is then snared and externalized through a contralateral sheath.

### Device release assessment

The 2 options available for the adequacy of pseudoaneurysm neck closure prerelease evaluation are cine angiography and Doppler ultrasound. A second catheter (pigtail and/or appropriate selective catheter) allows “prerelease” angiographic assessment of both; adequacy of pseudoaneurysm mouth occlusion and patency of adjacent vascular structures. Many authors have reported the utility of intracardiac echocardiography as an adjuvant to assess the adequacy of pseudoaneurysm neck closure using the color flow Doppler feature. Angiographic or Doppler evidence of residual flow should prompt a reevaluation of the pseudoaneurysm neck and a selection of an alternate device. Either technique appears to provide a satisfactory evaluation of pseudoaneurysm closure, but we found color Doppler to be more sensitive for residual flow detection. Operators familiar with both techniques will find complementary information when both technologies are utilized.

## Results

A total of 10 patients were found in our institution (2 women and 8 men with a mean age of 65.9 ± 12.9 years) compared to 40 patients in the literature (12 women, 27 men, and 1 unknown sex with a mean age of 64.1 ± 13.5 years) who had percutaneous AP intervention. Baseline patients’ characteristics and procedural details for the Loyola group are listed in Tables 1 and 2, respectively. Device delivery was successful in 92% (46) of all the cases, 100% of our cases, and 90% (36) of the literature. Two literature cases (5%) had a failure of successful device delivery. Of these 2 cases, 1 case resulted in inadequate positioning with attempted recapture complicated by hypotension, emergency sternotomy, and in-hospital death. In the other case, the procedure was stopped due to insufficient plug sizes, and the patient underwent surgical repair. Two other literature cases had missing data.

**Table 1:** Loyola patients’ demographic characteristics.

Patient	Age, y	Sex	CTD	Initial operation(s)	Time to diagnosis	Clinical presentation
1	68	Male	N/A	Mechanical AVR and ascending AD interposition graft, 2008	7 y	Incidental on CT
2	78	Male	N/A	CABG, 1983 and 1994	22 y	Incidental on CT
3	77	Male	N/A	AD repair, 2000	15 y	Hemoptysis and DOE
4	72	Male	N/A	Bioprosthetic AVR, aortic root replacement, and CABG, 2009	7 y	Follow-up imaging
5	59	Female	MS	Bioprosthetic AVR and ascending aortic repair, 2003	13 y	Follow-up imaging
6	71	Male	N/A	Aortic root replacement with valve conduit and reimplantation of coronary arteries, 2013	2 y	Hemoptysis
7	53	Male	EDS	Aortic dissection repair, 2011	4 y	Back and chest pain
8	76	Male	N/A	CABG, 1995	20 y	Chest pain
9	37	Female	N/A	Thoracic aortic aneurysm repair, 2015	1 y	Hemoptysis
10	68	Male	N/A	Thoracic AD and aneurysm repair, November 2018; aortic root dissection + aortic insufficiency repair and CABG, December 2018; descending AD bypass repair, February 2019	1 mo	Hypotension

This table illustrates the baseline demographic characteristics of all patients who underwent aortic pseudoaneurysm percutaneous repair.

AD, aortic dissection; AVR, aortic valve replacement; CABG, coronary artery bypass graft; CT, computed tomography; CTD, connective tissue disease; DOE, dyspnea on exertion; EDS, Ehlers Danlos syndrome; MS, Marfan syndrome; N/A, not available.

**Table 2:** Loyola patients’ procedural details.

Patient	AP location CT/MRI	AP size, mm, CT/MRI	Neck size, mm, angiography	AP sac size, mm, angiography	Guide/diagnostic catheter	Wire selection	Occluder device
1	Right lateral AA graft	37 × 41	6.3	51 × 38	7F Hockey stick	Benson wire, Amplatz SS	8 mm Muscular VSD
2	Anterior AA	72 × 55	6	78 × 75	5F SW 2, 6F JR4	Benson wire, Amplatz SS	22 mm Amplatzer ASD
3	Right lateral AA	83 **×** 56	2.2	33 × 25	6F JR4	Benson wire, Amplatz SS	18 mm Amplatzer ASD
4	Anterior wall aortic root	30 **×** 27	9.6	61 × 45	6F JR4	Amplatz SS	26 mm Amplatzer ASD
5	Anterior AA	29 **×** 27	11.9	50 × 27	6F JR4	Amplatz SS	16 mm Amplatzer ASD
6	Left aortic arch	68 **×** 80	5.9	62 × 89	6F IMA	Glidewire, Amplatz SS	16 mm Amplatzer ASD
7	Proximal DA	32 **×** 34	7.7	110 × 28	6F IMA	Glidewire	16 mm Vascular Plug
8	Site of SVG-RCA	90 **×** 80	2.5	33 × 64	6F multipurpose	Fielder FC	10 mm Vascular Plug
9	Aortic arch	36 **×** 11	3.1	76 × 50	6F multipurpose	Fielder FC	12 mm Amplatzer II Plug
10	AA	90	N/A	N/A	6F multipurpose/4F glide sheath	Fielder FC	8 mm Amplatzer Plug

This table shows the procedural details of all the patients who underwent percutaneous aortic pseudoaneurysm repair.

AA, ascending aorta; AP, aortic pseudoaneurysm; ASD, atrial septal defect; CT, computed tomography; DA, descending aorta; JR, Judkins Right; MRI, magnetic resonance imaging; N/A, not available; SVG-RCA, saphenous vein graft to right coronary artery; SW, sidewinder; VSD, ventricular septal defect.

We observed low rates of postprocedural complications. None of our patients developed renal insufficiency. None of our cases had major bleeding events. The literature group had 2 (5%) major bleeding complications, whereas 25 (62.5%) of its cases had no reported bleeding outcomes. The average length of stay was 1.9 (1-10) days in our group, but it was rarely reported in the literature group.

Clinical follow-up for 74% of all cases was 4 months (range of 2 days to 15 years). Follow-up was available for all our patients, ranging from 3 to 47 months (median follow-up 12 months, see Table 3). Follow-up was reported in only 27 (68%) literature cases with a median of 2 months (range of 2 days to 15 years). Imaging follow-up with CT or magnetic resonance imaging (MRI) was available for all our patients, but it was reported in only 17 cases (45%) of the literature group. AP sac size remained stable or had a reduction in size in 5 (50%) of our cases. AP percutaneous closure was performed in one of them to allow for the surgical excision of the AP sac, given its superior vena cava compression effect. Of the other 5 patients in our group, 1 died from chronic obstructive pulmonary disease exacerbation, and the remaining 4 (40%) required surgical repair for expanding AP sacs. In the literature group, 13 cases had worsening AP sac size, and 21 cases had no reported outcome. Four cases (10%) required surgical intervention in the literature group, including the 2 unsuccessful cases mentioned above. This difference can be attributed to our commitment to regular imaging postprocedural follow-up for the remainder of the patients’ lives.

**Table 3:** Loyola patients’ follow-up outcomes.

Patient	Follow-up duration, mo	Imaging modality	Imaging findings	Clinical status	Cause of death
1	17	CT (3 and 6 months)	Enlarging sac with persistent leak	Surgical pseudoaneurysm repair	N/A
2	12	CT (3 months and 1 year)	Stable size of pseudoaneurysm without leak	Deceased	COPD
3	34	CT (3, 12, and 34 months)	Enlarging sac suggesting leak	Surgical pseudoaneurysm repair	N/A
4	12	CT (3 months)	Enlarging sac with a small persistent leak	Surgical pseudoaneurysm repair	N/A
5	47	MR (3 and 6 months) CT (47 months)	Unchanged sac size	Clinically well	N/A
6	12	CT (3 months and 1 year)	Enlarging sac size	Coil embolization	N/A
7	12	MR (4 months) CT (12 months)	Stable sac size with a persistent small leak	Deceased	Ruptured AAA
8	3	CT (3 months)	Sac caused SVC syndrome-like symptoms	Surgical pseudoaneurysm resection	N/A
9	7	CT (3 and 6 months)	Stable size of pseudoaneurysm without leak	Deceased	Renal failure
10	4	CT (4 months)	Leak and increased collection size	Surgical pseudoaneurysm repair, deceased	Heart failure

This table shows the follow-up outcomes of all the patients who underwent percutaneous aortic pseudoaneurysm repair.

AAA, abdominal aortic aneurysm; COPD, chronic obstructive pulmonary disease; CT, computed tomography; MR, magnetic resonance; N/A, nonapplicable; SVC, superior vena cava.

In-hospital mortality occurred in 1 literature case and none in our group. All-cause mortality was observed in 6 (13%) of all reported cases (2 in the literature and 4 in the Loyola group). All Loyola group deaths were from non–AP-related causes (Table 3). Hospital cost for the percutaneous plug strategy can be attributed to a combination of physician salary, procedural expenses (lab time, staff wages, equipment, medications, and overheads) in addition to the cost of the hospital room, medications, laboratory testing, and radiology studies. We collected expense data within our tertiary care facility and estimated the combined cost to the hospital for a patient with a 3-night length of stay to be $12,978. The cost to the patient is approximately $49,580. There are no data available in the literature that outlines the hospital costs for surgical repair of an AP. However, a large review study conducted in the United States in 2016 found the mean complete cost for coronary artery bypass grafting (physician and hospital) was $151,271 (range $44,824 to $448,038).^32^ A summary of the outcomes is available in the Central Illustration and Table 4.

**Central Illustration:**
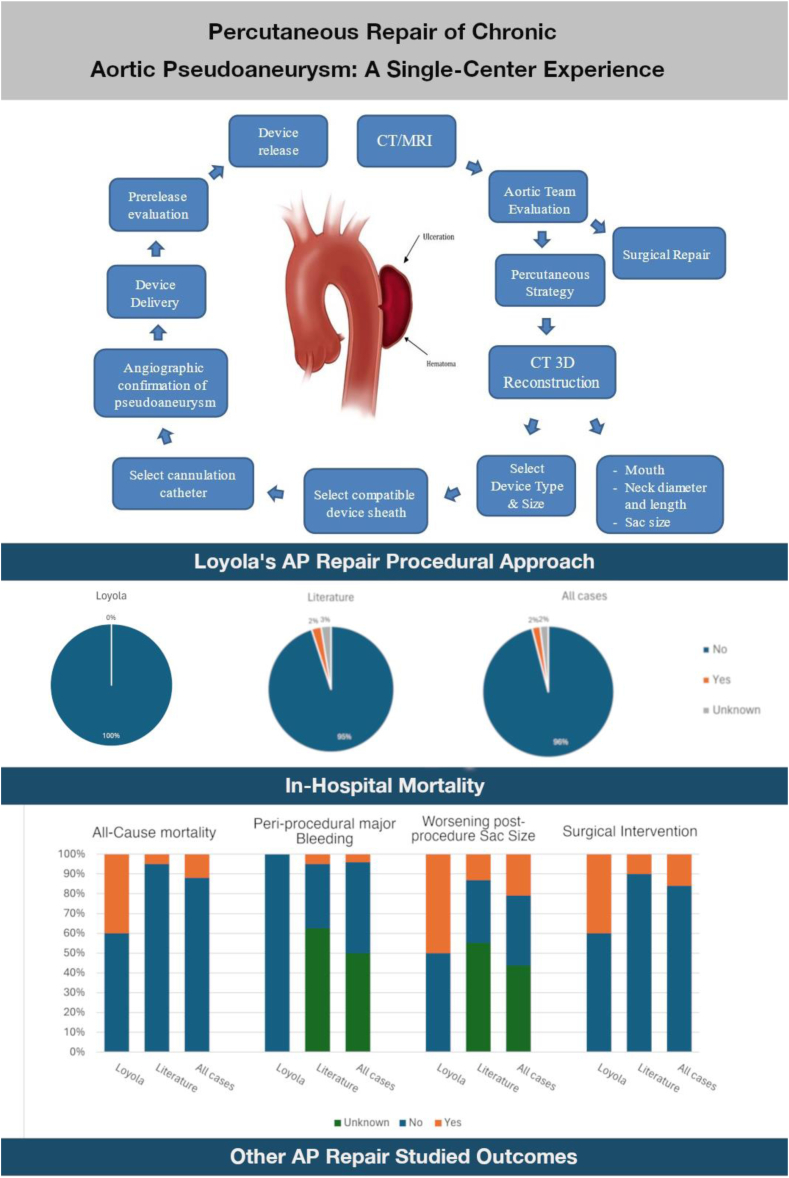
Untreated chronic aortic pseudoaneurysms (AP) have high mortality rates. Given significant perioperative complications, surgical or endovascular stent repairs cannot always be an option for AP repair. Percutaneous repair by vascular plugs can be a viable option for high-risk surgical candidates. We present our approach and experience with this technique along with the global literature experience. CT, computed tomography; MRI, magnetic resonance imaging.

**Table 4:** Reported outcomes of the literature and Loyola cases.

Outcome	All cases (N = 50)	Literature cases (n = 40)	LUMC cases (n = 10)
Successful device delivery, %	46 (92%)	36 (90%)	10 (100%)
Postprocedural major bleeding	2 (4%)	2 (5%)^a^	0 (0%)
Mean in-hospital length of stay duration, d (range)	–	Rarely reported	1.9 (1-10)
Median follow-up duration, mo (range)	–	2 (0.07-180)^b^	12 (3-47)
Worsening AP sac size rate, %	10 (20%)	5 (12.5%)^c^	5 (50%)
Surgical intervention rate, %	8 (16%)	4 (10%)	4 (40%)
In-hospital mortality rate, %	1 (2)%	1 (2.5%)^d^	0 (0%)
All-cause mortality rate, %	6 (12%)	2 (5%)^e^	4 (40%)^f^
Average hospital bill per case	–	Not reported	$49,580

This table shows the various outcomes of the AP repair cases in the literature and at the Loyola University Medical Center.

AP, aortic pseudoaneurysm; LUMC, Loyola University Medical Center.

a Twenty-five cases had no reported outcome.

b Only 13 cases had reported ≥3 months follow-up period.

c Twenty-one cases had no reported outcome.

d Another case had an unspecified mortality (in-hospital vs all-cause).

e Only 13 cases had reported ≥3 months follow-up period.

f All deaths were not AP-related.

## Discussion

Our report represents the largest analysis of chronic AP patients treated with a percutaneous plug/occluder strategy. The results of our review suggest that patients with AP formation can potentially be treated using a percutaneous plug/occluder approach with an acceptable expectation for initial success, with reasonable short and long-term morbidity and mortality. When compared to traditional open repair, the utilization of a percutaneous approach offers several potential advantages. We found our average length of stay to be 1.9 days (range 1-10 days) with an estimated hospital cost of approximately $13,000. On average, Medicare/insurance was billed approximately $50,000 for the percutaneous repair, which we hypothesize to be less than the cost associated with open surgical repair, given the complexity of an open procedure, in addition to the intensive care unit days, anesthesia, and operating room support. As mentioned previously, surgical repair of pseudoaneurysm carries with it anywhere from 6.9% to 12.6% in-hospital mortality. This is compared to our patient cohort in whom we saw no in-hospital mortality, and only 1 mortality case was observed in the literature group.

Limitations of this study include its retrospective design. Reporting bias favoring the publication of individual cases with favorable outcomes presents a real concern when evaluating procedural success and procedural complications. Individual cases with unsuccessful delivery or unacceptable outcomes are unlikely to be submitted for publication. Although small, our local experience includes all patients treated percutaneously at our institution and should provide reasonable insight into outcome expectations for chronic AP patients treated with plug/occluder devices. A direct comparison of surgical and percutaneous outcomes is handicapped by the lack of an adequate and reliable comparison of the preprocedure patient characteristics. Patients referred for percutaneous pseudoaneurysm repair may have technical considerations or clinical comorbidities, which put those patients at higher risk for open surgical repair and selection for a higher acuity population in the percutaneously treated patient population. The high 30-day mortality seen with endograft repair reported in the literature is likely explained by this selection bias. We used this review of the literature to describe the gravity of pseudoaneurysm detection and highlight the serious consequences of current approaches to this vexing clinical problem. It is our feeling that a summary of the global experience is a more realistic presentation of pseudoaneurysms management. Our purpose is not to compare the percutaneous strategy to surgical repair in favorable surgical candidates but to offer a “bailout strategy” for poor surgical candidates.

Long-term follow-up is incomplete and variable in the publications reviewed. The relatively recent adaptation of the percutaneous approach by our group limits the duration of our long-term follow-up in this report. Follow-up reported in the literature is variable but frequently is limited to a single repeat image which again hampers the conclusion on the durability of the technique. Results from our review support a more dedicated investigation in the form of a registry-based design with the use of a historical control or a prospective head-to-head trial comparing open pseudoaneurysm repair and this percutaneous approach.

The value of dedicated imaging (CT/MRI) and a commitment to the reconstruction of each patient's anatomy cannot be underestimated. Our experience has recognized the importance of dedicated CT and MRI imaging with 3D reconstruction as a major point of emphasis. The advantage of measurement of the mouth of the pseudoaneurysm could improve the accuracy required for device selection and therefore improve procedural success. Preprocedure reconstructions can further shorten procedure times and potentially reduce procedural costs by limiting the undersizing of the device with the required subsequent removal and reinsertion of a larger, appropriately sized device. Follow-up imaging is important in the chronic management of aortic disease. Timing the follow-up CT scans in the management of AP is not standardized, but these patients clearly require some degree of postprocedure surveillance.

As one can see from the literature, incomplete closure, sac enlargement, and new very delayed pseudoaneurysm formation can be detected on routine monitoring. Surveillance strategies must weigh the risks of sequential and cumulative radiation and contrast exposure against the consequences of undetected aortic pathology.

## Conclusion

Results suggest that patients can safely and effectively be treated with an interventional approach to AP repair. It is our feeling that these complex cases should be performed in centers that have made a commitment to the treatment of aortic pathology. Experienced surgeons with a focus on contemporary approaches to aortic and vascular repair form the foundation of such programs. Interventional cardiologists and vascular surgeons with broad experience in the use of vascular occluders, structural heart occlusion devices, and vascular occlusion devices are critical to maximizing safety and procedural success in patients treated with a percutaneous approach.

## Perspectives

The literature clearly documents the natural history of postsurgical PA in terms of morbidity, mortality, and cost. Limitations of the traditional surgical and endovascular techniques are also well-described. The description of the application of percutaneous approaches is limited in the literature. Our manuscript adds to the understanding of the procedure through the introduction of 3D printing and long-term follow-up. A national registry to track patient characteristics, procedural techniques with early outcomes, and long-term imaging follow-up would contribute to our understanding of the technology.
